# The Physiological Action of Picolinic Acid in the Human Brain

**DOI:** 10.4137/ijtr.s2469

**Published:** 2009-04-28

**Authors:** R.S. Grant, S.E. Coggan, G.A. Smythe

**Affiliations:** 1 School of Medical Sciences, Faculty of Medicine, University of New South Wales, Sydney NSW, 2052; 2 Australasian Research Institute, Sydney Adventist Hospital, 185 Fox Valley Rd., Wahroonga, NSW, 2076; 3 Bioanalytical Mass Spectrometry Facility (BMSF), UNSW, Sydney, NSW 2052

## Abstract

Picolinic Acid is an endogenous metabolite of L-tryptophan (TRP) that has been reported to possess a wide range of neuroprotective, immunological, and anti-proliferative affects within the body. However the salient physiological function of this molecule is yet to be established. The synthesis of picolinic acid as a product of the kynurenine pathway (KP) suggests that, similar to other KP metabolites, picolinic acid may play a role in the pathogenesis of inflammatory disorders within the CNS and possibly other organs.

In this paper we review the limited body of literature dealing with the physiological actions of picolinic acid in the CNS and its associated synthesis via the kynurenine pathway in health and disease. Discrepancies and gaps in our current knowledge of picolinic acid are identified highlighting areas of research to promote a more complete understanding of its endogenous function in the brain.

## Background

**Figure 1.**
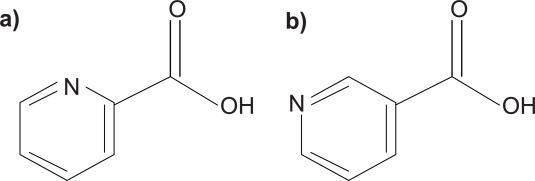
Chemical structures of the isomers Picolinic acid **a**) and Nicotinic acid **b**).

Picolinic Acid (PIC) is a six-member ring structure compound (Fig. 1) that has been detected in a variety of biological mediums including, cell culture supernatants, blood serum^1^ cerebrospinal fluid (CSF),^2^ human milk, pancreatic juice and intestinal homogenates.^3^

**Figure 2.**
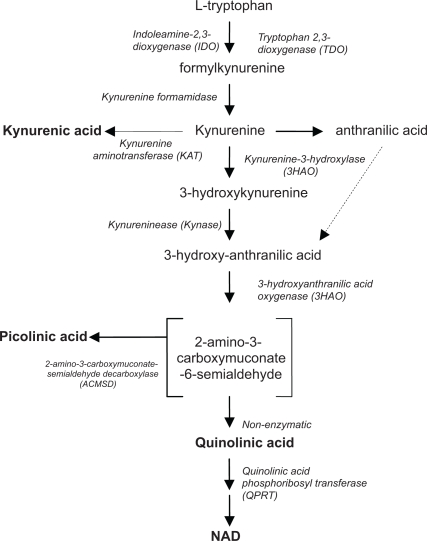
The kynurenine pathway in the CNS.

PIC is synthesised from L-tryptophan (TRP) via a sequent side branch of the kynurenine pathway (KP) involving enzymatic shunting of an aminocarboxysemialdehyde intermediate toward PIC over non-enzymatic synthesis of the neurotoxin quinolinic acid (QUIN) (Fig. 2). TRP catabolism via the KP reputedly accounts for greater than 95% of the daily TRP turnover in the CNS.^4^ While a clear understanding of the physiological role of various KP metabolites and overall pathway dynamics remains elusive, at least a portion of TRP is converted to the essential pyridine nucleotide, nicotinamide adenine dinucleotide (NAD).^5^,^6^

Alan Mehler^7^ was the first to postulate that PIC was a metabolic product of the KP. Having identified that an enzymatic reaction perturbed the formation of PIC away from the synthesis of QUIN, Mehler questioned whether; a) PIC was a normal metabolic product of TRP with some physiological function and b) PIC production had some effect on NAD synthesis. While research has confirmed that PIC is a metabolite of the KP and knowledge of other aspects of the pathway has grown considerably over recent years, the salient endogenous function of PIC remains elusive, with its effect on NAD metabolism and significance in the CNS yet to be determined.

Alterations in KP metabolism have been implicated in the pathophysiology of a variety of CNS inflammatory diseases including; Alzheimer’s disease,^8^–^10^ Multiple Sclerosis,^11^ Parkinson’s,^12^ Cerebral Malaria,^13^ Amyotropic lateral sclerosis^14^ and HIV infection.^15^ It is therefore reasonable to consider whether changes in PIC levels also correlate with either cellular or clinical pathology in any of these conditions.

In this review we discuss the potential physiological action of PIC identified by previous studies and place these findings within the context of both relevance to physiological conditions and associated KP metabolism. Discrepancies and gaps in our current knowledge of PIC are identified and areas of research that may promote a more complete understanding of PIC’s endogenous function are suggested.

## Physical Properties of PIC

Picolinic acid is a six-membered ring structure and isomer of nicotinic acid, containing five carbon atoms a nitrogen and a carboxyl group at position 2.

A number of synonyms exist for picolinic acid including, Pyridine-2-carboxylic Acid; 2-Pyridinecarboxylic acid; o-Pyridinecarboxylic Acid and alpha-Pyridinecarboxylic Acid. As a pure crystal, PIC has a melting point at ∼137 °C and is soluble in water to 887 g/l. The partial molal volume of PIC at infinite dilution is 83.8 ml mole, calculated from the density of its aqueous solutions. The refractivity at infinite dilultion is 31.9 ml mole. Picolinic acid exists mainly in the zwitterion form in solution and displays relatively high viscosities where a 48% solution has about the same viscosity as a 38% sucrose solution at the same temperature.^16^

The most widely researched physical characteristic of picolinic acid is its efficient chelator properties. Chelator activity for PIC was first reported by Weidel in 1879, where PIC was shown to efficiently chelate both copper and iron. Later Suzuki et al^17^ in 1957 reported its efficient chelation of a range of metals including Ni, Zn, Cd, Pb and Cu.

Capitalizing on its chelation properties PIC-metal complexes are now widely used as a means of introducing bioactive metals into biological systems. In particular, as the element chromium appears to play a role in carbohydrate and lipid metabolism, dietary supplementation with chromium picolinate has been advocated in type 2 diabetes.^18^ As chromium is not assimilated particularly well from the diet more effective absorption is achieved through the ingestion of a PIC-chromium chelate. Chromium picolinate [Cr(pic)(3)] supplementation reportedly has effects on blood glucose and lipid metabolism and body composition.^18^ In these formulations PIC is generally considered the non-active ingredient that helps solubilise the metal through the formation of the chelate complex.

## Biological Synthesis of Picolinic Acid

TRP can be metabolised through oxidative degradation via the kynurenine pathway (KP) to one of three main end products; kynurenic acid (KYNA), PIC and NAD (Fig. 2). The KP begins with the oxidative cleavage of the amino acid tryptophan by either of the two enzymes *indoleamine 2,3-dioxygenase* (IDO; EC 1.13.11.17) or *tryptophan 2,3-dioxygenase* also called *tryptophan pyrolase* (TDO; EC 1.13.11.11) to produce formylkynurenine (Fig. 2). Both IDO and TDO are haem-requiring enzymes and are considered rate limiting for this pathway.

TDO is found predominantly in mammalian liver and can be induced by a number of factors including fasting, glucocorticoids, hydrocortisone, L-tryptophan and nicotinic acid.^19^,^20^ In contrast IDO is found mainly in extrahepatic tissues including brain, placenta, spleen, lung, kidney, alimentary tract and epididymis.^20^,^21^ Unlike TDO, IDO does not contain an activating site for tryptophan analogues^22^ and is induced primarily by the proinflammatory cytokine IFN-γ. IDO also uses the reactive oxygen intermediate (ROI) superoxide as opposed to molecular oxygen as a cofactor.^22^ TDO also differs from IDO in regard to substrate specificity. TDO uses L-tryptophan exclusively as substrate whereas IDO can metabolise both L and D-tryptophan as well as serotonin and other related indoleamines.^23^

In the process of PIC synthesis tryptophan is catabolised through kynurenine to 3-hydroxyanthranilic acid. This is then further acted upon by the enzyme *3-hydroxyanthranilic acid oxygenase* (3HAO; EC 1.13.11.6), an enzyme present in both cytosol and synaptosomal fractions^24^ to produce the intermediate 2-amino-3-carboxymuconic semialdehyde (Fig. 2).

The rate limiting enzyme for PIC production *amino-ß-carboxymuconate-semialdehyde-decarboxylase* (ACMSD; EC 4.1.1.45) will preferentially convert this intermediate to 2-aminomuconic semialdehyde with subsequent non-enzymatic conversion to picolinic acid.^25^ Non enzymatic rearrangement of the intermediate occurs when ACMSD is saturated with substrate^26^ allowing the production of quinolinic acid (QUIN). It has been suggested that under normal conditions these two pathways control equal flux.^27^ ACMSD is therefore a key enzyme directing KP metabolism towards PIC production, and is expressed at a ratio of 1300 : 30 : 1 in kidney, liver and brain, respectively.^28^ Not surprisingly the activity of ACMSD has been shown to be inversely proportional to the amount of NAD synthesised from tryptophan.^25^

## Physiological Role of KP Metabolism

In the periphery, the role of the KP appears to be primarily directed toward the production of NAD, with other KP metabolites excreted unchanged in high concentrations in the urine.^29^ In the CNS, however, the purpose of KP activation is less clear. Generation of NAD is integral to brain function and may be derived *de novo* from tryptophan.^6^ However, KP activation potentially occurs in all brain cell types, including neurons,^10^ astrocytes,^30^ infiltrating macrophages,^31^ dendritic cells^32^ and blood brain barrier (BBB) endothelial cells.^33^ With many kynurenines (the collective term for KP metabolites) showing neuro-modulatory function in the CNS it is unclear at what point synthesis and release of various kynurenines change from being part of normal physiological processes to becoming contributors to pathophysiological activity.

Quinolinic acid (QUIN) and KYNA are two neuroactive KP metabolites that have received considerable attention for their modulation of the excitatory amino acid N-methyl-D-aspartate (NMDA) receptor. While QUIN shows neurotoxic effects by over activation of the NMDA receptor, KYNA offers neuro-protection by blocking receptor function via an allosteric glycine site.^34^ Emphasis has, therefore, been placed upon the importance of maintaining a balanced ratio between these two metabolites.

Often overlooked however, is the observation that PIC also shows antagonistic properties towards the toxic effects of QUIN via an unknown mechanism.^35^,^36^ PIC’s role in maintaining the balance between neurotoxic and neuro-protective KP metabolites requires further investigation.

## Experimental Actions of PIC: *In vitro* and *In vivo*

On its own, in experimental systems, PIC is reported to elicit a number of potential effects within the body, particularly involving immune function and antimicrobial activity.

*In vitro* studies suggest that PIC (at supernatant concentrations of 1–4 mM) can enhance macrophage effector functions through the enhancement of interferon-γ (INFγ) dependant nitric oxide synthase (NOS) gene expression^37^,^38^ and induce expression of the macrophage inflammatory proteins (MIP)1α and 1β.^39^ While the mechanism producing the synergism with IFN-γ is not known PIC mediated induction of MIP 1α and β is thought to be through an iron chelation dependant process.

High PIC concentrations (1–4 mM) have also been reported to selectively inhibit a variety of viruses in culture including the Human Immunodeficiency virus (HIV), Herpes Simplex virus (HSV), and Simian virus (SV) in culture.^40^,^41^ PIC appears to produce its antiviral activity through an initial cytotoxic action which in turn increases apoptosis of infected cells and a reduction in viral replication.^41^ PIC in combination with IFNγ has also been shown to inhibit retroviral expression of the J2 retrovirus,^42^ again through an unknown mechanism.

Anti-microbial effects of PIC (2.5–40 mM) have been observed against *Mycobacterium avium* complex (MAC), with significant enhancement of the antimicrobial action of the drugs clarithromycin, rifampin and various fluoroquinolones.^43^,^44^ It is suggested that as PA efficiently chelates metal ions, such as Zn^2+^ and Fe^2+^ it is therefore likely that its antimicrobial activity against MAC organisms is due to its ability to chelate essential metal ions such as Fe^2+^.^43^

Some investigators have also observed an effect of PIC on tumour growth. *In vivo* studies on mice inoculated with MBL-2 lymphoma cells showed that those treated with injections of PIC (100 mg/Kg) in combination with activated macrophages, had significant increases in lifespan compared to control.^45^ These effects are arguably due to macrophage activation mechanisms through IFN-γ mediated mechanisms as suggested previously.^45^

Importantly high concentrations of PIC, relative to physiological levels, were used in all of the investigations discussed above. Tables 1 and 2 highlight the large discrepancy between PIC concentrations used experimentally *in vitro/in vivo* (Table 1) and reported levels of endogenous PIC (Table 2). With PIC concentrations *in vivo* generally ranging within the low to mid *nano* (10^−9^) molar range the milimolar (10^−3^) concentrations used in the above cited studies have generally resulted in an approximately one million fold difference between physiological and experimental PIC concentrations. While these studies do indicate possible therapeutic roles for PIC, the large discrepancy between endogenous and experimental PIC levels does raise a question over the relevance of these observations to the natural physiological function of PIC.

## PIC Concentrations in Diseases of the CNS

An often useful aid to understanding the physiological role of an uncharacterised biological molecule is the study of changes in the endogenous levels of the molecule in health and disease. Current literature in this area is however limited to a report by Medana et al.^13^ showing raised PIC levels in the CSF of patients with cerebral malaria and a recent report from our own group in which no significant difference in PIC levels were identified between different CNS disease categories.^46^ Importantly we have shown that any attempt at correlating PIC levels in the CNS may be complicated by an apparent diurnal fluctuation in CSF PIC levels as discussed later in this review.

As PIC is part of a more complex pathway, it is relevant to review how other kynurenines are affected by disease and under what conditions altered levels have been observed (for summary see Table 3).

It has been suggested that altered KP metabolism contributes significantly to the pathophysiology of neurodegenerative and inflammatory disorders of the CNS.^34^ Lower levels of KYNA and the enzymes involved in its production have been found in the plasma, CSF and erythrocytes of Parkinson’s and Alzheimer’s disease sufferers.^9^,^47^,^48^ On the other hand, Huntington’s disease subjects exhibited elevated cortical QUIN levels in the early stages of disease onset, suggesting excitotoxicity as the cause of later neurodegeneration.^49^

Various viral infections show an up regulation of the entire KP. HIV infection exhibits increased levels of both QUIN and TRP in the human brain,^50^ while in the CSF, QUIN has been further correlated to worsening brain atrophy.^51^ Poliovirus infected macaques showed increases of CSF QUIN, KYNA, L-kynurenine, kynurenine-3-hydroxylase, and kynureninase activities, with the magnitude of increase correlating with the severity of observed motor deficits.^52^

In a study of African children suffering from cerebral malaria elevated PIC and QUIN concentrations predicted a fatal outcome to the disease.^13^ These observations are consistent with results reported in an animal model using centrally infected (*Plasmodium berghei*) mice, where CSF PIC also increased markedly with malarial infection.^53^

While some studies have shown that immune activation can result in elevated PIC levels^37^–^39^ a recent study by our group failed to find any correlation between CSF PIC concentrations and CNS disease states.^46^ This data was generated from a population of 241 patients that were suspected of meningitis as well as other nervous system disorders. Immune activation markers such as white cell count and C-reactive protein also did not show any correlation with CSF PIC concentrations.

These results led us to investigate whether other biological factors may influence the production of PIC in the CNS.

## Biological Factors Influencing PIC Levels in the CNS

We have recently reported that CSF PIC levels in the CNS may be influenced by both the age of the subject and time of sample collection.^46^ This observation has not been previously reported, although other studies have noted similar patterns for other kynurenines. In a population without detectable neurological disease Kepplinger et al.^54^ and Heyes et al.^9^ observed that CSF KYNA levels significantly increased with advancing age. Consistent with our observation, the activity of the PIC producing enzyme ACMSD has been shown to increase with age in studies of rat kidney, liver and small intestine.^55^ Unfortunately, an investigation of age-associated changes in ACMSD activity in human tissue is yet to be reported.

From a sample of subjects with no apparent CNS disease, we observed that CSF PIC concentrations display a significant diurnal variation depending on the time of sample collection.^46^ Importantly, QUIN levels did not show this diurnal pattern, thus making the observation unique to PIC alone and suggesting the diurnal variation in ACMSD activity is most likely caused by substrate availability. Further, this circadian fluctuation was not observed in CSF PIC levels from patients with apparent CNS disease, suggesting that this temporal rhythm in PIC concentrations can be significantly perturbed during times of immune activation.

This observed diurnal fluctuation is remarkably similar to a serial CSF sampling study of 12 healthy volunteers by Kennedy and colleagues.^56^ A significant diurnal pattern in CSF TRP metabolism was observed, with low TRP levels at near noon, and a maximum reached at 11 pm—midnight.^56^ From this, it was proposed that the peak to trough availability of TRP as a precursor molecule may be of sufficient magnitude to influence other metabolite processes, such as melatonin secretion, which also follows a diurnal cycle.^56^

With a diurnal fluctuation modelled by variations in TRP levels, it can be proposed that the availability of TRP in the CSF has a direct affect on substrate availability for CNS PIC production.

Future studies are required to establish what conditions affect the baseline diurnal fluctuation of CSF PIC and what pathophysiological/disease states may be linked to these changes.

## Enzymatic Control of PIC Production: ACMSD

As the availability of substrate will impact the synthesis of PIC in the CNS,^56^ it is relevant to consider whether the activity of surrounding metabolic enzymes could also influence PIC levels.

ACMSD activity will be influenced by the availability of its relevant substrate which in turn is dependant on flux through the KP. The activity of IDO, the rate limiting enzyme of the KP, (Fig. 2) is therefore positioned to significantly influence the synthesis of the PIC precursor.

IDO activity has been shown to be involved in a variety of physiological processes. IDO activation can influence maternal tolerance toward the allogenic fetus^57^ regulate autoimmune disorders,^58^ and suppress transplant rejection.^59^ Inhibitors of this enzyme are, therefore, currently being developed for cancer immunotherapy.^60^

While the mechanism through which IDO displays its immune activity appears to involve tryptophan concentrations in the microenvironment^57^ the influence of downstream metabolites such as PIC on these and other activities has not been extensively investigated.

As ACMSD substrate required for PIC synthesis is dependant on IDO activity, any therapeutic manipulation of IDO will affect the synthesis of PIC. Understanding the pathophysiological role of PIC may help prevent potential PIC associated side effects following IDO inhibition.

The position of ACMSD within the KP establishes it as the rate limiting enzyme for PIC synthesis (Fig. 2). Interestingly, a number of nutritional factors and hormonal effects have been observed to alter the activity of this enzyme.

High protein diets, the diabetic condition^61^ and increased glucocorticoids^62^ have all been demonstrated to increase the activity of ACMSD. Whereas high levels of dietary polyunsaturated fatty acids,^63^ peroxisome-proliferators^64^ and the environmental plasticizer contaminants, phthalate esters^65^ appear to significantly down-regulate the activity of this enzyme.

While the physiological rationale for these changes is not known, high protein diets have previously been shown to also increase enzymes associated with energy metabolism.^66^ Is it possible that ACMSD/PIC may play a role in the regulation glucose/energy metabolism? It is relevant to note that a number of metallopicolinate complexes have been found with insulinomimetic activity where the presence of the picolinate significantly enhances the insulin sensitising activity.^67^

Importantly most studies in this area have measured the activity of ACMSD from the peripheral tissue of animal models. Peripheral tissue data may or may not correlate substantially with the centrally located human protein which apparently has a relatively low level of expression.^28^ The lack of relevant experimental data relating to the human CNS highlights the need for future research in this area.

## PIC and the BBB

The measurement of PIC in the CSF is assumed to reflect endogenous PIC production within the CNS alone. However it is also possible that CSF levels may be influenced by transfer of PIC from the peripheral circulation.

Chromium picolinate is an established dietary supplement used to treat conditions of insulin resistance.^18^ The ability of this complex to produce affects within the CNS has been queried.^68^ Aggett et al.^69^ reported that divalent metal complexes of PIC do not readily pass through lipid bilayers. PIC itself also has a relatively low partition coefficient (logP = 0.098^a^) reflecting its low solubility in a lipophilic medium. This makes it unlikely that PIC would pass through the BBB on its own. In addition, Smythe and colleagues^8^ reported that PIC plasma levels were at concentrations 3–15 times higher than those in the brain tissue and CSF (Table 2). For such a concentration ratio to be maintained, it is thought that limited flux of PIC occurs between the CNS and the periphery.

While the above observations indicate that CSF PIC levels reflect PIC synthesis within the CNS, other KP metabolites may contribute significantly to their respective cerebral pools.

In experiments using *in situ* brain perfusion techniques on rats, L-kynurenine and 3-hydroxykynurenine were found to be taken up into the brain at significant rates across the BBB via the large amino acid transporter.^70^ Owe-Young and colleagues^33^ recently found that KP activation occurs in BBB endothelial cells, and when treated with INFγ, increased the expression of kynurenine but not QUIN or PIC. That these metabolites contribute to PIC production through increased substrate availability has been proposed but never tested.^71^ It would be beneficial to our understanding in this area if future studies examined the concentration of PIC in the CNS under conditions of both peripheral KP supplementation and peripheral KP activation.

In summary, there are a number of biological factors that can potentially affect PIC levels and synthesis in the CNS including; age and circadian rhythms and hormonal and nutritional factors.

However the physiological role(s) of PIC within the CNS is not well understood. Improved understanding may be achieved by characterizing the changes to cell/organ biochemistry/physiology at PIC concentrations within acceptable physiological concetrations. In addition, it has not been established whether there is a link between PIC concentration and any disease state. Whether PIC production is as clearly connected to pathophysiological changes as other kynurenines such as QUIN and KYNA requires further investigation with careful attention to the confounding effects of age and diurnal rhythm.

## Figures and Tables

**Table 1.** PIC concentrations used experimentally.

**Reported physiological effects of PIC**	**[PIC] used**	**Reference**
Induces MIP 1 α & β expression^1^	4000 μM	39
Enhances IFN-γ mediated NO production^1^	4000 μM	37
Anti-tumour activity^2^	100 mg/kg	45
Anti-viral: SV-transformed cells^1^	1000–3000 μM	40
Anti-viral: HIV infected cells^1^	1500–3000 μM	40
Anti-viral: J2 retrovirus^1^	4000 μM	42
Anti-microbial: against MAC	2500–20000 μM	43
complex^1^	2500–40000 μM	44

Experiment performed using;

1 cell culture,

2 whole animal (mouse).

**Table 2.** Endogenous concentrations of PIC.

**Reported endogenous PIC locations**	**[PIC] (μM)**	**Reference**
Plasma	0.299 +/− 0.034	8
Brain (cortical tissue)	0.100–0.150	8
CSF (no brain injury)	0.017 +/− 0.005	8

**Table 3.** Altered Kynurenine pathway metabolites in disease.

**CNS disease**	**Alteration of kynurenines in the CNS (CSF/brain tissue^Ψ^)**			**Reference**
	**QUIN**	**KYNA**	**PIC**	
Alzheimer’s	nt	nt	n/s^Ψ^	8
	nt	**↓**	nt	9
Parkinson’s	nt	**↓**	nt	12
Multiple sclerosis	**↑**^Ψ^	**↓**	nt	11
Huntington’s	**↑**	nt	nt	49
Cerebral Malaria	**↑**	**↑**	**↑**	13
HIV infection	nt	**↑**	nt	52
Down Syndrome	nt	**↑**^Ψ^	nt	72
Amyotrophic	nt	**↑**	nt	73
lateral sclerosis	**↑**	nt	nt	14

**↑** = Elevated levels compared to control,

**↓** = decreased levels compared to control.

**Abbreviations:** nt, not tested, n/s, not significant.

## Footnotes

a Log P calculated by Advanced Chemical Development log P Batch version 100 software.
